# Atrial fibrillation among women in endurance sports: sex-specific risk and mechanisms

**DOI:** 10.1007/s00421-026-06433-0

**Published:** 2026-09-19

**Authors:** Solange Piccolo, Alessandro Sarai, Gaia Giuriato, Gianluigi Dorelli, Luca Tomasi, Roberto Scarsini, Flavio Luciano Ribichini, Federico Schena, Giacomo Mugnai, Massimo Venturelli

**Affiliations:** 1 Division of Cardiology, Department of Medicine, University of Verona, Verona, Italy; 2 Department of Theoretical and Applied Sciences, eCampus University, Novedrate, Italy; 3 Department of Neurosciences, Biomedicine and Movement Sciences Verona, University of Verona, Strada Le Grazie 8, Room 3.15 Istituti Biologici 3, 37134 Verona, Italy; 4 Division of Cardiology, Integrated University Hospital of Verona, Verona, Italy; 5 Department of Internal Medicine, University of Utah, Salt Lake City, UT USA

**Keywords:** Atrial fibrillation, Exercise, Endurance, Female, Sex-factors, Sports

## Abstract

Regular physical activity remains a cornerstone of cardiovascular health; however, prolonged, high-volume endurance training may paradoxically increase the risk of atrial fibrillation (AF). While this association is well established in male population, sex-specific evidence in women is still limited, despite their rapidly growing participation in endurance sport. Epidemiological studies consistently demonstrate a J-shaped association between exercise exposure and AF relative risk in both sexes. Women appear to have a broader protective window, tolerating substantially higher exercise doses (estimated up to ~83 MET-h/week in cohort studies, equivalent to approximately 10–12 h of moderate-to-vigorous endurance training per week) before AF risk begins to rise, whereas men show a risk increase at lower exposure levels. At the extreme end of the spectrum, elite female endurance athletes may also experience elevated AF relative risk, confirming that the upper inflection of the J-curve applies to both sexes. Mechanistic evidence indicate that atrial structural remodelling occurs similarly in men and women, yet sex hormones and autonomic tone appear to modulate arrhythmogenic vulnerability. Overall, current evidence supports sex-aware clinical counselling, promoting the cardiovascular benefits of endurance exercise in women while considering potential risk at very high training loads.

## Introduction

Regular physical activity is a cornerstone of cardiovascular health, as emphasized by the current European Society of Cardiology (ESC) guidelines (Visseren et al. 2021), capable of reducing morbidity and mortality and contributing to psychophysical well-being. Moderate aerobic exercise confers a cardiovascular protective effect, also reducing the risk, burden, and severity of atrial fibrillation (AF) (Buckley et al. 2025). However, a prolonged, high volume of endurance training can paradoxically increase this risk (ESC n.d.; Van et al. 2024). Endurance athletes exposed to chronically high exercise volumes face a 3- to 8-fold higher risk of developing AF, with a prevalence reaching up to 15% among veteran elite competitors (Molina et al. 2008; Grimsmo et al. 2010).

For many years, this association between intense endurance sport and AF has been well documented in male athletes (Molina et al. 2008; Guasch and Mont 2015; Hoogsteen and Schep 2004) and evidence regarding women remains limited. Historically lower female participation and their underrepresentation in sports science research have contributed to a substantial knowledge gap. Therefore, a key question for clinical practice is whether women experience the same training-induced adaptations, follow the same dose–response curve of exercise exposure, and should be counselled using the same exercise prescription thresholds as men. Understanding these sex-specific aspects is essential to optimize clinical decision-making and to prevent AF without undermining the clear health benefits of exercise.

## Epidemiological insights

Atrial fibrillation is the most common sustained arrhythmia worldwide, associated with significant morbidity, mortality, and healthcare costs (Joglar et al. 2023). Although women have a lower age-adjusted incidence and prevalence of AF compared to men, their longer life expectancy results in a similar overall burden (Ko et al. 2016). Moreover, women with AF experience more severe complications, with a higher risk of thromboembolism (Cove et al. 2013), a poorer quality of life, and a worse overall prognosis (Zylla et al. 2016).

Physical inactivity is a well-recognized risk factor for AF (Van et al. 2024; Christophersen and Ellinor 2016; Kannel et al. 1998), but surprisingly, physical activity acts as both a protective and a risk factor, describing a peculiar J-shaped curve (Newman et al. 2021; Stergiou and Duncan 2018). AF is the most prevalent arrhythmia among athletes (Newman et al. 2021); exercise-induced AF is usually diagnosed in middle-aged patients with a long history of elevated volumes of endurance sports (Molina et al. 2008; Grimsmo et al. 2010; Andersen et al. 2013; Guasch and Mont 2017).

The ESC guidelines (Visseren et al. 2021) recommend at least 150–300 min per week of moderate-intensity aerobic physical activity or 75–150 min of vigorous intensity to reduce cardiovascular events, but no recommendation exists for an upper limit, especially regarding the total volume of the exercise.

Sex is increasingly recognized as a factor influencing the clinical features and mechanisms of AF, yet women, especially athletes, remain underrepresented in the scientific literature (Ose et al. 2025; Hunter et al. 2024). Most studies include up to 90% men, leaving a significant data gap on AF risk in female athletes (Stergiou and Duncan 2018; Wilhelm et al. 2011).

**Fig. 1:**
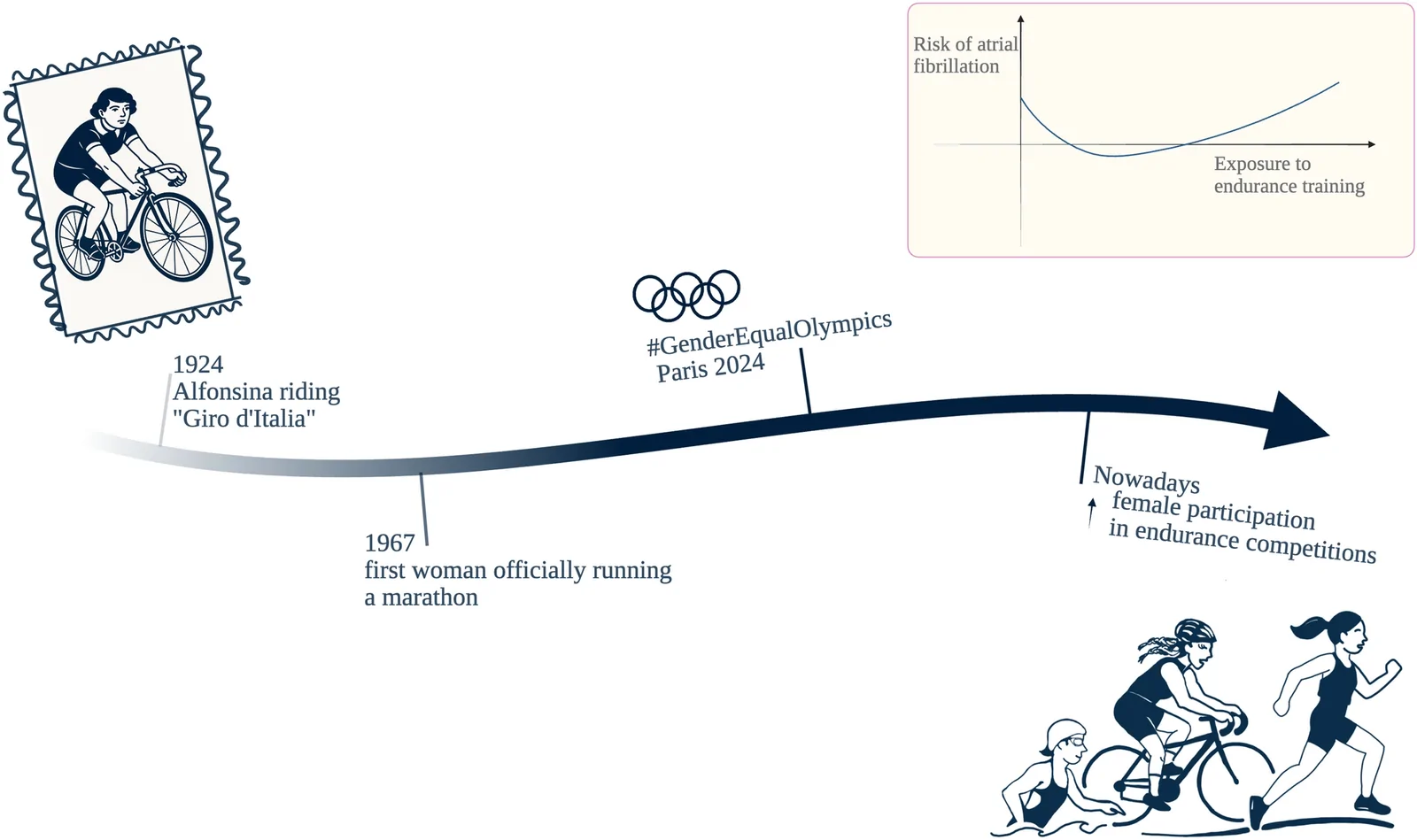
Illustrative timeline highlighting key milestones in women’s participation in endurance sports: From Alfonsina Strada in 1924 “Giro d’Italia” to Kathrine Switzer as the first official marathon female participant in 1967, women have only recently gained access to endurance competition. The inset depicts the characteristic J-shaped relationship between endurance exercise exposure and atrial fibrillation risk, a pattern now also observed among female athletes. Illustrative timeline depicting the historical progression of women’s participation in endurance sports, highlighting key milestones and increases in female involvement over time

Female participation in endurance sports has increased substantially in recent decades Fig. 1, reaching almost 50% of total race participants and full gender parity at the Paris 2024 Olympic Games. However, representation progressively decreases with increasing race distance, particularly in ultra-endurance disciplines (27% at IRONMAN® Hawaii; <10% in Triple/Deca Iron) (Knechtle et al. 2023; Lepers et al. 2013; Loosli et al. 2025; Rüst et al. 2014), contributing to the persistent scarcity of sex-specific research in endurance sport.

## Current studies on atrial fibrillation in female athletes

**Fig. 2:**
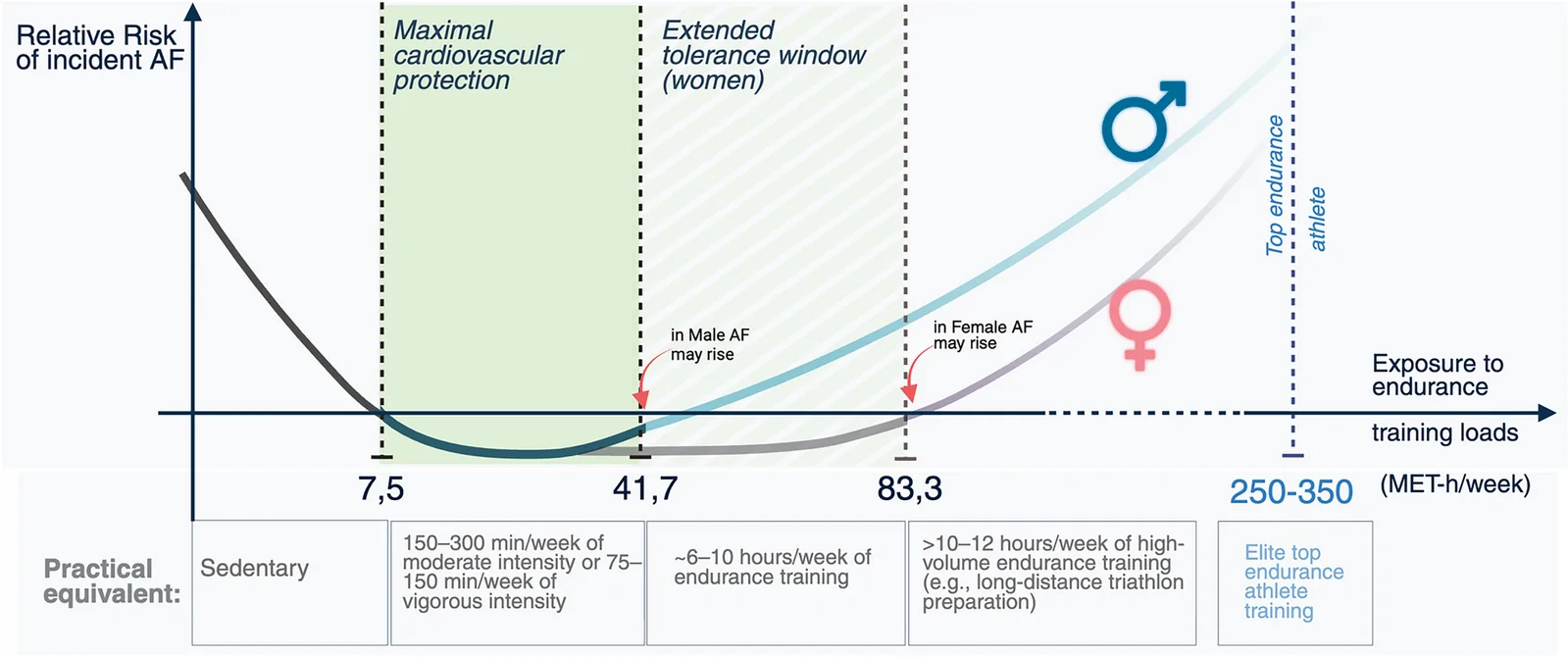
J-shaped curve of atrial fibrillation relative risk in male and female. Comparison between the j-shape curve of atrial fibrillation relative risk and training load in male and in female, showing a wider tolerance window in women. Sex-specific relationships between endurance training load and atrial fibrillation risk. A J-shaped curve compares male and female risk trajectories, showing a wider tolerance window in women. Icons depict endurance sports, and a conceptual triangle summarizes trigger, substrate, and modulating mechanisms. The exercise thresholds displayed in the figure are primarily based on the UK Biobank study by Elliott et al. Reported values originally expressed as MET-min/week were converted to MET-h/week (÷ 60) to ensure consistency with the units adopted throughout this review and to facilitate comparison across studies. Practical exercise equivalents are approximate and depend on exercise intensity

Emerging evidence suggests a complex, sex-specific interaction between exercise and AF risk. As early as 2016, a meta-analysis suggested a protective effect of physical activity in women, albeit with high heterogeneity among studies (Mohanty et al. 2016). Large-scale studies have helped clarify the shape of the dose–response curve. The Women’s Health Study (Everett et al. 2011), involving over 34,000 women, showed that an exercise volume of approximately 7.5 MET-h/week (roughly equivalent to the minimum recommended level of aerobic physical activity, e.g. ~150 min/week of moderate-intensity exercise) was associated with a 14% lower risk of incident AF compared with less active women., supporting the protective effect at the lower end of the J-shaped curve Fig. 2. The Tromsø Study (Morseth et al. 2016), a prospective study with a sex-balanced cohort, confirmed a J-shaped trend in both sexes but noted that while a small increase in AF risk was observed in very active women; it was not statistically significant, unlike in men who had a higher absolute risk at all activity levels. The most compelling evidence of a wider "safety margin" for women comes from the UK Biobank (Elliott et al. 2020) cohort study. Across more than 500,000 individuals, men showed an AF risk increase above approximately 2500 MET-min/week (≈41.7 MET·h/week, corresponding to approximately 5–7 h/week of moderate-to-vigorous endurance exercise at an average intensity of 6–8 METs), whereas in women the threshold was approximately twice as high (5000 MET-min/week, ≈83.3 MET·h/week, equivalent to approximately 10–14 h/week at a comparable exercise intensity).

Overall, these findings suggest that women can tolerate higher exercise doses before their AF risk increases, even beyond current guideline recommendations. However, it is important to note that exercise exposure is commonly expressed as MET-h or-min/week, an integrated measure of exercise dose that combines exercise intensity and duration. Although useful for standardizing comparisons across studies, it does not disentangle the relative contributions of exercise intensity, training volume, and other determinants of physiological training load. In fact, despite the utility of MET for comparing exercise dose, actual training loads in endurance disciplines vary enormously across sports, training intensity distribution and time of the season. For example, recreational or sub-elite marathon runners may accumulate an estimated 40–100 MET-h/week, committed cyclists or triathletes may exceed 100–150 MET-h/week during heavy training phases, and the training load of a world-class triathlete may reach up to an estimated 250–300 MET h/week(Cejuela and Sellés-Pérez 2022; Falk Neto et al. 2021; Mujika 2017; Dasa et al. 2024).

Nevertheless, the upper end of this dose–response relationship also deserves consideration. A recent retrospective matched cohort study by Drca et al. (2023) compared top-level Swedish female endurance athletes with the general population. The results confirmed the upper end of the J-shaped curve for women as well: elite female endurance athletes had a significantly increased risk of AF compared to controls. This crucial study demonstrates that while women may have a higher threshold, prolonged endurance training increases AF risk in this population too. Finally, a study by D’Ambrosio et al. (2025), provided an important mechanistic insight, showing that male sex increased the odds of AF by 4-fold, independent of the presence of atrial ectopic triggers or fibrosis. This strongly suggests that the risk difference lies not so much in the structural substrate but in sex-specific biological modulating factors, as represented in Table 1.

**Table 1:** Key epidemiological studies on sex differences in exercise-induced atrial fibrillation

Study & year	Cohort & design	Key Finding in Women	Key findings in men / comparison	Implications
Morseth et al. (2016) (Tromsø Study)	20,484 adults (49.7% women), prospective cohort	A J-shaped risk curve was observed, but the risk increase in the most active group was not statistically significant	Higher absolute risk of AF at all activity levels compared to women	Suggests a similar J-shaped trend but with a lower absolute risk in women
Everett et al. (2011) (Women’s Health Study)	>34,000 women, prospective cohort	Moderate exercise (7.5 MET-h/week) is associated with a 14% lower risk of AF incidence	N/A (female-only cohort)	Confirms the protective effect of moderate activity in women (left side of the J-curve)
Elliott et al. (2020) (UK Biobank)	502,543 individuals (52.5% women), prospective cohort	Pronounced and consistent protective effect up to 83.3 MET-h/week. 8–16% risk reduction at 8–42 MET-h/week	Modest risk reduction up to 38 MET-h/week; vigorous activity is associated with a 12% *increase* in risk	Women have a significantly wider "therapeutic window" for exercise before risk increases
Drca et al. (2023)	228 elite Swedish female endurance athletes vs. 1368 matched controls, retrospective cohort	Elite female athletes had a significantly increased risk of AF (HR 3.67, 95% CI 1.71−7.87, adjusted)	N/A (female-only cohort, but conceptually comparable to studies on male athletes)	Confirms that the upper end of the J-curve also exists for women at extreme levels of endurance training
D’Ambrosio et al. (2025)	Cohort of former endurance athletes (male and female)	N/A (focus on sex as a predictor)	Male sex independently increased the odds of AF by 4-fold, despite no differences in triggers or fibrosis	Suggests the risk difference is driven by sex-linked biological factors beyond structural remodelling

*AF* atrial fibrillation, *HR* hazard ratio, *CI* confidence interval, *MET* metabolic equivalent of task

## Pathophysiology: underlying mechanism and sex differences

The mechanisms leading to AF in athletes are multifactorial, involving both mechanical and electrical atrial remodeling.

**Fig. 3:**
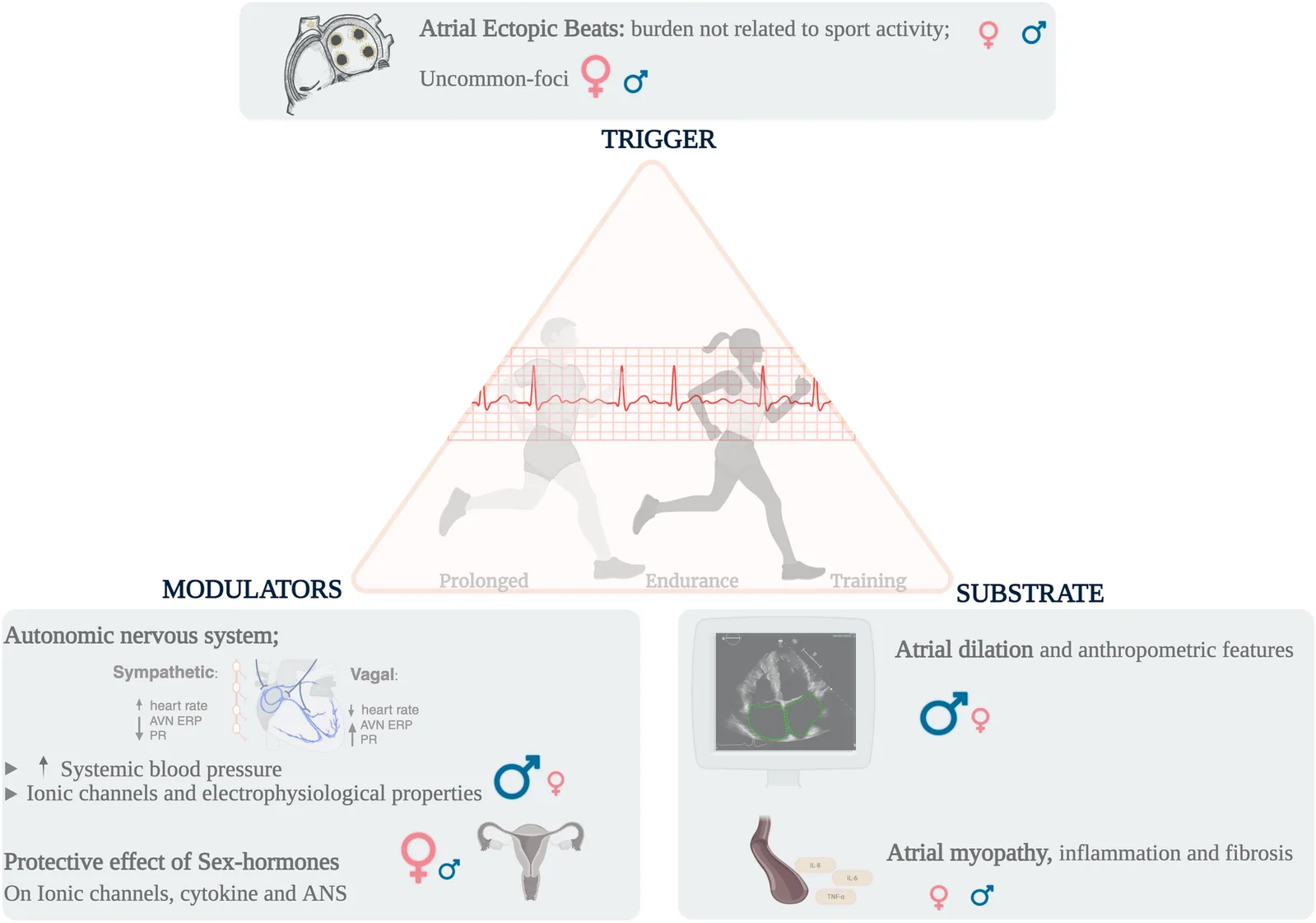
Conceptual representation of atrial fibrillation mechanisms in endurance athletes. The model integrates structural, electrophysiological, and hormonal factors contributing to sex-specific susceptibility

Accordingly, AF is thought to result from the interaction between three fundamental components: (i) an arrhythmogenic substrate (defined as the structural and electrophysiological alterations that create a permissive environment for AF initiation and maintenance), (ii) triggering ectopic activity, and (iii) modulatory factors such as autonomic tone and sex hormones (that determine the susceptibility of the substrate to sustain the arrhythmia), Fig. 3.

Current evidence suggests that sex differences in these mechanisms lie more in the factors that modulate the arrhythmogenic substrate rather than in the substrate itself (Guasch and Mont 2017; D’Ambrosio et al. 2025) as represented in Table 2**.**

**Table 2:** Pathophysiological mechanisms of atrial fibrillation in athletes: a sex-based comparison

Factor	Category	Observations in male athletes	Observations in female athletes	Postulated sex-specific difference & implication
Atrial size & structure	Substrate	Significant bi-atrial enlargement. (Hsieh et al. 2025)	Significant bi-atrial enlargement, but less pronounced compared to males (Mosèn et al. 2014; D’Ascenzi et al. 2014)	Women may exhibit a distinct baseline atrial phenotype (smaller atrial mass, lower atrial voltage, smaller cardiomyocytes) (McManus et al. 2010; Nakashima et al. 2026 . Whether it confers greater resilience to exercise-induced remodelling is unknown
Atrial fibrosis	Substrate	Animal models show fibrosis development with intense training (Benito et al. 2011; Alcarraz et al. 2025). And higher incidence of electrocardiographic markers associated with myocardial fibrosis. (Kramer et al. 2024)	No significant difference in the prevalence of LGE compared to males in one study (D’Ambrosio et al. 2025)	Histopathological evidence suggest that fibrotic substrate may develop similarly for both sexes in general population (Nakashima et al. 2026) , suggesting it is not the primary driver of the risk difference. The key may be how modulators act on this substrate
Inflammation	Substrate / Modulator	Elevated inflammatory cytokines (IL-6, TNF-α) post-exercise (Weight et al. 1991; Bernecker et al. 2013; Stumpf et al. 2016)	Post-intense exercise elevation of inflammatory cytokines was also observed (Zar et al. 2016; Janikowska et al. 2020). Levels may be influenced by the menstrual cycle (O’Brien et al. 2007)	While both sexes experience exercise-induced inflammation, the cyclical hormonal environment in women may alter the chronic inflammatory state, with unclear net effects on arrhythmogenesis
Autonomic tone	Modulator	More pronounced vagal dominance: greater bradycardia, sinus pauses, longer PR interval. Vagal triggers are common (Skov et al. 2016; Guasch et al. 2013)	Less pronounced vagal remodelling compared to men(Skov et al. 2016; Dart et al. 2002). Autonomic function is influenced by the menstrual cycle (Yildirir et al. 2002)	The more extreme vagal tone in men could be a key pro-arrhythmic factor, shortening atrial refractory periods and facilitating re-entry electric circuits
Sex hormones	Modulator	Knowledge gap: exercise-specific effect on arrhythmogenesis	Oestrogen may prolong atrial APD (anti-arrhythmic) and has cardioprotective effects on remodelling and fibrosis in preclinical studies (Liu et al. 1998; Drici et al. 1996; Buitrago et al. 2000; Jovanovǐ et al. 2000). No direct evidence in female athletes	The hormonal environment is a prime candidate to explain the risk disparity. Oestrogen appears protective, while testosterone may be pro-arrhythmic (Tran et al. 2024), directly altering atrial electrophysiology
Ectopic triggers (PABs)	Trigger	PAB burden is not significantly higher than in controls (Cipriani et al. 2020)	No sex difference in overall PAB prevalence. However, women are more susceptible to non-pulmonary vein foci (e.g., superior vena cava) (Lee et al. 2005; Tai et al. 2000)	The overall trigger burden appears similar, reinforcing the idea that the difference lies in the susceptibility of the substrate (modulation) rather than the triggers themselves

*RA* right atria, *LGE* late gadolinium enhancement, *APD* action potential duration, *PAB* premature atrial beat

### The arrhythmogenic substrate: structural remodeling and fibrosis

Harmonic atrial enlargement, proportional to ventricular remodeling, is a hallmark of the “athlete’s heart” (Vaziri et al. 1994) in both sexes (D’Ascenzi et al. 2014; Hsieh et al. 2025). Women generally have lower baseline atrial volumes and wall thickness than men (McManus et al. 2010).Atrial dilation represents a chronic adaptation to endurance training, and is not, per se, associated with an increased risk of atrial fibrillation (Trivedi et al. 2020). Rather, it is the atrial contractile dysfunction, or atrial myopathy, resulting from repetitive wall stress that creates the substrate (Trivedi et al. 2020). Indeed, the greater atrial fibroblast reactivity makes the atria a susceptible chamber to haemodynamic overload (Burstein et al. 2008). In this context, preclinical mechanistic studies in male Wistar rats have consistently demonstrated atrial dilatation, collagen deposition, fibrosis, and increased arrhythmia inducibility following prolonged vigorous endurance training. (Benito et al. 2011; Alcarraz et al. 2025). Prolonged exposure to endurance training has also been associated with a higher likelihood of late gadolinium enhancement on cardiac magnetic resonance (Wilson et al. 2011), with no sex differences observed (D’Ambrosio et al. 2025), suggesting the fibrotic substrate may develop similarly in both sexes. Systemic inflammation, another consequence of high-intensity exercise, is closely linked to myocardial fibrosis (Guo et al. 2012). A sustained systemic inflammatory state and elevated circulating cytokines are additional consequences of prolonged, high-intensity exercise (Alcarraz et al. 2025; Weight et al. 1991; Bernecker et al. 2013; Müssigbrodt et al. 2017). One study (Stumpf et al. 2016) involving 25 professional football players reported both left atrial remodelling and sustained increases in cytokines (IL-6, TNF-α, IL-8), although the sex of the athletes was not specified. Similar cytokine elevations have also been observed specifically in female athletes (Zar et al. 2016; Janikowska et al. 2020), while C-reactive (Khera et al. 2005) protein and fibroblast growth factor-23 (Mathew et al. 2014), have been found to be higher in women than men in general epidemiological studies. Notably, cytokine levels in women may also be influenced by the menstrual cycle (O’Brien et al. 2007).

Recent evidence from Yamaguchi et al. (Nakashima et al. 2026) also suggests that sex differences in the atrial substrate may exist even before exercise-induced remodelling. Women exhibited lower atrial voltage on electroanatomical mapping than men, a finding that the authors hypothesized could be related to their inherently smaller atrial mass and cardiomyocyte size. Interestingly, despite these electrophysiological differences, no significant sex differences were observed in histopathological parameters, including intercellular space, fibrosis, myofibrillar loss, myocardial nuclear density, cardiomyocyte size, or amyloid deposition, in patients with AF (Nakashima et al. 2026). Whether this sex-specific baseline atrial phenotype influences the adaptive response to long-term endurance exercise and the subsequent risk of AF remains unknown.

### Sex-specific modulators: autonomic tone and hormones

If the structural substrate can be similar, the factors modulating its electrophysiological properties appear to be the main drivers of the risk disparity (Herrera et al. 2025). Exercise may also influence arrhythmogenic modulators and the electrophysiological properties of the atria. Athletes are particularly prone to vagally-dominant situations, which can reduce heart rate and the cardiac conduction, non-uniformly shorten the atrial refractory period and alter ionic channel-currents (Patterson et al. 2005; Oliveira et al. 2011), overall facilitating arrhythmias and AF ((Skov et al. 2016; Guasch et al. 2013)). Male endurance athletes showed a higher blood pressure (Wilhelm et al. 2011), more bradycardia, pauses and longer PR interval compared to female athletes (D’Ambrosio et al. 2025). Notably, the menstrual cycle can also influence the cardiac effects of the autonomic nervous system (Dart et al. 2002); during the luteal phase, a more pronounced bradycardia was observed compared to follicular phase (Yildirir et al. 2002).

Sex hormones represent the most obvious sex-specific modulator (Nakamura et al. 2007; Nakagawa et al. 2006). Estrogens have been shown to exert cardioprotective effects, modulating cardiac ion currents to prolong the atrial action potential duration, a potentially anti-arrhythmic effect (Liu et al. 1998; Drici et al. 1996; Buitrago et al. 2000). In animal models, estradiol has been shown to modulate potassium currents and calcium fluxes, conferring protection against hypoxia-reoxygenation injury (Buitrago et al. 2000; Jovanovǐ et al. 2000). A rabbit model has also demonstrated sex-related differences in the electrophysiological characteristics of the pulmonary veins and left atrium, with a higher susceptibility for trigger activity and automaticity in males (Tsai et al. 2011). Therefore, the potential role of female sex hormones as modulators of arrhythmias is intriguing; however, the direct involvement of both oestrogens and progesterone in the pathogenesis of atrial fibrillation remains largely unclear. Regarding the levels of testosterone, the current evidence is relatively sparse and inconclusive (Hu et al. 2022), suggesting it may rather represent a risk factor, particularly in older adults (Tran et al. 2024).

Moreover, although genetic predisposition may act as a risk-modifying factor, no specific genetic mutations, especially related to sex, have been consistently identified in athletes with AF to date (Ko et al. 2016). Eventually, ectopic triggers, such as premature atrial beats (PABs), are necessary to initiate the arrhythmia. Conversely, their prevalence does not appear to be higher in endurance athletes compared to sedentary individuals, and no sex differences in their overall burden have been observed. This further strengthens the hypothesis that the risk difference lies not in the trigger, but in the susceptibility of the atrial substrate, which is strongly influenced by the sex-specific hormonal and autonomic environment. Of note, the burden of PABs is not higher in endurance athletes compared to sedentary individuals, and it appears to depend more on age than on the intensity or volume of endurance exercise (Cipriani et al. 2020). Still, women seem to be more susceptible than men to uncommon-foci PABs, with female sex being the sole independent predictor of superior vena cava ectopy ((Lee et al. 2005; Tai et al. 2000)).

## Clinical implications and future directions

Women have historically been underrepresented in both endurance sports and sports science research, but recent trends indicate a positive shift toward sex inclusion (Hunter et al. 2024; Devries and Jakobi 2021; Joyner et al. 2025). Current evidence supports a dose-dependent relationship between exercise and AF; while regular moderate physical activity is protective in both sexes and recommended by current cardiovascular guidelines (Visseren et al. 2021), women appear to tolerate higher cumulative exercise doses before AF risk increases. Nevertheless, at the highest levels of endurance training, elite female athletes also exhibit an increased risk of AF (Table 3 and Fig. 2).

**Table 3:** Zones of relative risk for incident atrial fibrillation and exposure to training

Category	MET-h/week	Practical equivalent	Clinical interpretation
Recommended physical activity in general population	7.5 MET -h/week*	150–300 min/week of moderate intensity or 75–150 min/week of vigorous intensity physical activity (Visseren et al. 2021)	Maximum cardiovascular protection, lowest AF risk
High-risk zone (men)	> 41,7 MET h/week*	≈ 5–7 h/week of vigorous endurance training	Increased AF relative risk observed beyond this threshold
Extended low-risk range (women)	Up to 83.3 MET-h/week*	≈ 5–10 h/week of vigorous endurance training	Protective effect generally maintained; no significant AF relative risk increases documented
Potential high-risk zone (women)	> 83.3 MET h/week*	>10–12 h/week of high-volume endurance training (e.g., long-distance triathlon preparation)	Possible transition into the upper end of the J-curve; AF relative risk may rise at extreme volumes

*MET* metabolic equivalent of task, *AF* atrial fibrillation

^*^The exercise thresholds are primarily based on the UK Biobank (Elliott et al. 2020). MET-h/week values were obtained by converting MET-min/week values reported in the original studies (÷ 60) to provide a uniform unit across studies. Practical equivalents are approximate and may vary according to exercise intensity, sport discipline and training characteristics

Importantly, the increase in AF risk reported in the studies refers to a relative risk and not an absolute probability of developing the condition. The reason for the sex-disparity in AF risk seems to lie more in modulating factors acting on the arrhythmic substrate, such as autonomic tone and sex hormones, rather than in differences in the substrate itself or in ectopic triggers. Indeed, most available studies involve premenopausal women, leaving several key questions unanswered.

First, the hormonal profile of participants is often unreported, despite the potentially significant role of estrogens. Future studies should include longitudinal designs that monitor female athletes across multiple menstrual cycles.

Second, it remains unclear whether high volumes of endurance training during the reproductive years have long-term implications for AF risk after menopause, particularly considering that early-onset AF (< 50 years) is more prevalent in men (Kalstø et al. 2024). ), whereas women generally accumulate major cardiovascular risk factors and AF-related comorbidities later in life (Ko et al. 2016; Magnussen et al. 2017) . Although this temporal difference may contribute to the longer period during which the cardioprotective effects of exercise are maintained in women, its interaction with lifelong endurance training remains unknown. Therefore, ageing should be considered when interpreting the relationship between exercise exposure and AF risk, particularly in longitudinal studies spanning the menopausal transition, as outlined in Table 4. Finally, current exercise prescription guidelines are still largely based on evidence from male-dominated cohorts.

**Table 4:** Knowledge gaps and future research directives on atrial fibrillation and female athletes

Identified knowledge gap	Specific research question	Proposed study design	Potential impact on clinical practice
Impact of menstrual cycle and hormonal fluctuations	Do transient hormonal shifts during the menstrual cycle create "windows of vulnerability" by altering autonomic tone, QT intervals, or atrial ectopy burden in female endurance athletes?	Longitudinal cohort study of elite premenopausal athletes with dense sampling: daily hormone assays (salivary/urinary), continuous ECG monitoring (Holter/patch), and daily training logs over multiple cycles	Could lead to cycle-based training recommendations or risk stratification, especially for athletes with known arrhythmias
Long-term post-menopausal risk	Does a lifetime of high-volume endurance training accelerate post-menopausal AF risk, or does the pre-menopausal protective effect confer a lasting benefit?	Long-term follow-up of existing female athlete cohorts (e.g., the Drca et al. cohort) into their 6th and 7th decades, tracking AF incidence and comparing it to matched sedentary controls	Informs long-term cardiovascular risk counselling for aging female athletes
Hormone replacement therapy (HRT)	What is the effect of different HRT formulations on AF risk in former elite female athletes?	Nested case–control study within a large population-based registry, comparing AF incidence among post-menopausal former athletes on various HRT regimens versus those not on HRT	Provides evidence-based guidance for HRT prescription in this unique patient population
Defining the upper limit of "safe" exercise	Can a sex-specific upper threshold of training volume/intensity (in MET-hours/week) be defined beyond which AF risk begins to significantly increase for women?	Large-scale prospective cohort studies with granular, validated physical activity tracking (wearables + diaries) in both male and female master athletes, powered to detect the upper inflection point of the J-curve	Would allow for the development of the first evidence-based guidelines on the upper limits of exercise for AF prevention, tailored by sex

*AF* atrial fibrillation, *ECG* electrocardiogram, *MET* metabolic equivalent of task

### Perspective

**Fig. 4:**
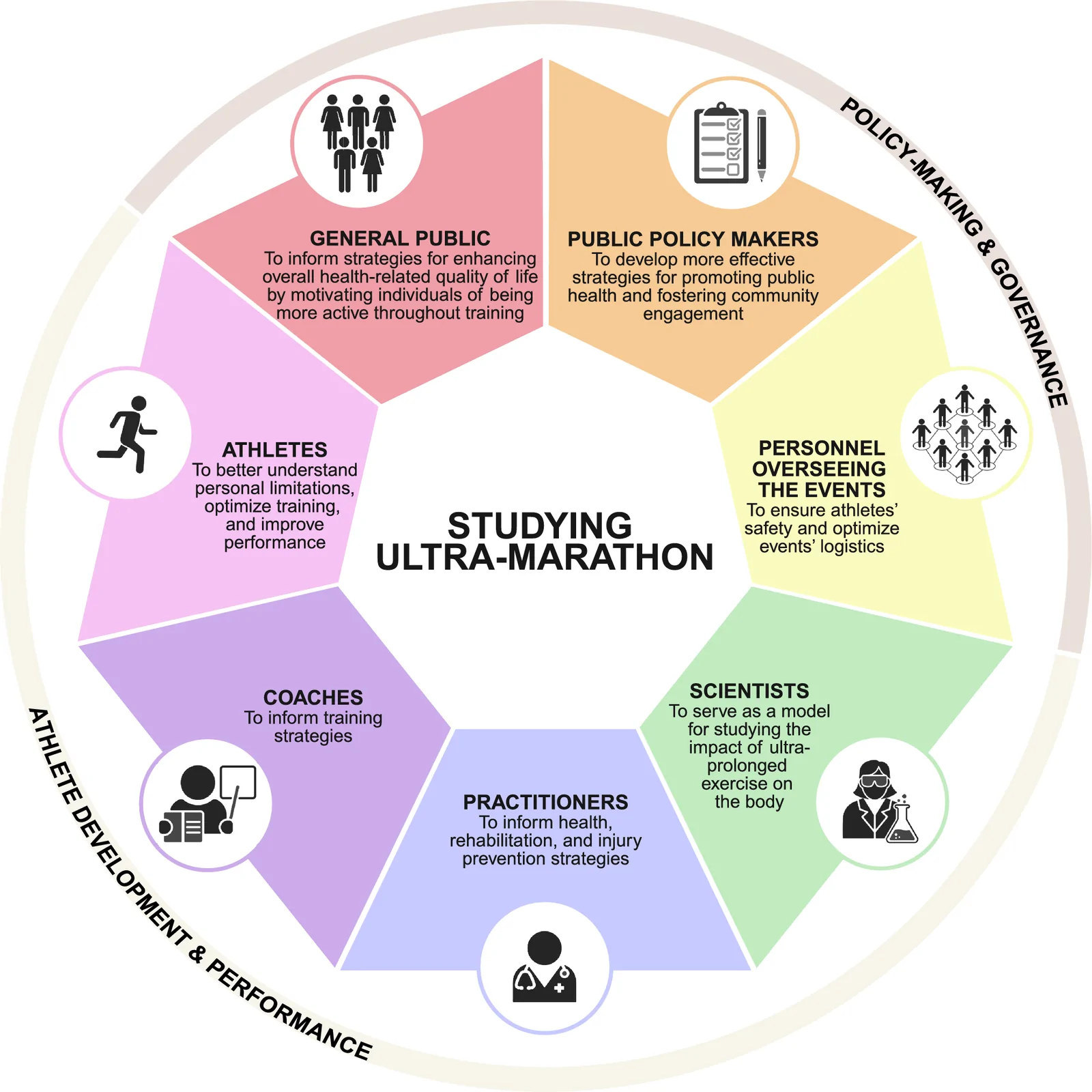
Conceptual framework illustrating the multi-level relevance of studying ultra-endurance exercise, with specific implications for atrial fibrillation risk and mechanisms in female athletes. Ultra-marathon settings are presented as a physiological model to investigate the effects of prolonged exercise on cardiovascular function and remodeling. The figure highlights key stakeholders including scientists, coaches, athletes, practitioners, event personnel, policymakers, and the general public and their respective roles in translating evidence into practice. These perspectives collectively contribute to athlete development and performance, health promotion, safety optimization, and governance strategies. In the context of women in endurance sports, this framework underscores the need to integrate sex-specific considerations into research, training, and clinical practice to better understand and mitigate atrial fibrillation risk

The paradoxical dose–response relationship between endurance training load and cardiovascular health has largely been derived from male populations, leaving important gaps regarding sex-specific responses and real-world training exposure. This narrative review integrates evidence from exercise physiology, cardiovascular adaptation and epidemiology to suggest that women engaged in endurance exercise exhibit a wider tolerance window to training loads before the relative risk of AF begins to rise. As sports cardiology moves toward more inclusive and stratified research designs and a more sex-aware approach, individualised counselling that accounts for sex-specific physiological differences and risk profiles may help prevent AF without undermining the substantial cardiovascular and systemic benefits of physical activity. As represented in Fig. 4 a conceptual framework positions ultra-endurance exercise as a translational model to investigate cardiovascular adaptations and maladaptation, with relevance to atrial fibrillation in female athletes. It integrates multiple levels of influence, from biological mechanisms (e.g., cardiac remodelling, autonomic imbalance, and inflammation) to applied contexts involving athletes, coaches, clinicians, and policymakers. The model emphasizes how prolonged exercise exposure may differentially affect women, highlighting the need for sex-specific approaches in both research and practice. By bridging mechanistic insights with real-world stakeholders, the framework underscores the importance of translating evidence into targeted strategies for performance optimization, risk stratification, and prevention of atrial fibrillation in endurance-trained women.

## Data Availability

No new data were created or analysed in this study.

## Declarations

### Patient involvement

No.
